# Effect of methylsulfonylmethane on oxidative stress and *CYP3A93* expression in fetal horse liver cells

**DOI:** 10.5713/ajas.20.0061

**Published:** 2020-08-24

**Authors:** Kyoung Hwan Kim, Jeong-Woong Park, Young Mok Yang, Ki-Duk Song, Byung-Wook Cho

**Affiliations:** 1 Department of Animal Science, College of Natural Resources and Life Sciences, Pusan National University, Miryang 50463, Korea; 2 Life and Industry Convergence Research Institute, Pusan National University, Miryang 50463, Korea; 3 Department of Pathology, School of Medicine, Institute of Biomedical Science and Technology, Konkuk University, Chungju 27478, Korea; 4 Department of Agriculture Convergence Technology, Jeonbuk National University, Jeonju 54896, Korea

**Keywords:** *CYP3A* Family Genes, Horse Fetal Liver Cells, Oxidative Stress, Methylsulfonylmethane, Antioxidant

## Abstract

**Objective:**

Stress-induced cytotoxicity caused by xenobiotics and endogenous metabolites induces the production of reactive oxygen species and often results in damage to cellular components such as DNA, proteins, and lipids. The cytochrome P450 (CYP) family of enzymes are most abundant in hepatocytes, where they play key roles in regulating cellular stress responses. We aimed to determine the effects of the antioxidant compound, methylsulfonylmethane (MSM), on oxidative stress response, and study the cytochrome P450 family 3 subfamily A (*CYP3A*) gene expression in fetal horse hepatocytes.

**Methods:**

The expression of hepatocyte markers and *CYP3A* family genes (*CYP3A89*, *CYP3A93*, *CYP3A94*, *CYP3A95*, *CYP3A96*, and *CYP3A97*) were assessed in different organ tissues of the horse and fetal horse liver-derived cells (FHLCs) using quantitative reverse transcription polymerase chain reaction. To elucidate the antioxidant effects of MSM on FHLCs, cell viability, levels of oxidative markers, and gene expression of *CYP3A* were investigated in H_2_O_2_-induced oxidative stress in the presence and absence of MSM.

**Results:**

FHLCs exhibited features of liver cells and simultaneously maintained the typical genetic characteristics of normal liver tissue; however, the expression profiles of some liver markers and *CYP3A* genes, except that of *CYP3A93*, were different. The expression of *CYP3A93* specifically increased after the addition of H_2_O_2_ to the culture medium. MSM treatment reduced oxidative stress as well as the expression of *CYP3A93* and heme oxygenase 1, an oxidative marker in FHLCs.

**Conclusion:**

MSM could reduce oxidative stress and hepatotoxicity in FHLCs by altering *CYP3A93* expression and related signaling pathways.

## INTRODUCTION

The liver plays a vital role in detoxification of xenobiotics such as carcinogens, environmental toxins, and drugs. The cytochrome P450 (CYP) family is a superfamily of heme-containing monooxygenases involved in the metabolism of approximately 80% of all drugs, xenobiotics, and endogenous metabolites in the liver [1]. *CYP* genes are classified into families and subfamilies according to their amino acid sequence identities. Enzymes that share more than 40% sequence identity belong to the same family, whereas those that share more than 55% sequence identity belong to the same subfamily [2]. CYP1, CYP2, and CYP3 are involved in the metabolism of numerous compounds and their expression is influenced by various factors such as xenobiotics, cytokines, and hormones, as well as genetics, disease, sex, and age [3]. *CYP3A* is expressed in many tissues, including the liver, intestines, gastric system, kidneys, lungs, adrenal glands, olfactory system, skin, prostate, and brain [4]. Functional studies have shown that each *CYP3A* isoform functions in a substrate-specific manner [5,6]. In the *CYP3A* subfamily, CYP3A4 is the most abundant isoform found in hepatocytes and plays a key role in the metabolism of drugs including steroids [7]. Several *CYP3A* genes have been annotated in the horse genome, for example, *CYP3A89*, *CYP3A93*, *CYP3A94*, *CYP3A95*, *CYP3A96*, and *CYP3A97*, which exhibit high amino acid similarities with the human *CYP3A4* gene [8]. However, there is limited information on the involvement of horse CYP3As in substrate metabolism.

Reactive oxygen species (ROS), for example, hydrogen peroxide (H_2_O_2_), hydroxyl radicals, and superoxide anions that are generated by CYP metabolic processes, can induce and intensify oxidative stress in the liver [9]. The rate of H_2_O_2_ generation varies depending on the intrinsic properties of each CYP enzyme [10]. Excess ROS not only mediate cellular toxicity but also activate endogenous antioxidant mechanisms [11]. One of these endogenous mechanisms involves the translocation of NF-E2-related factor 2 (NRF2) from the cytosol to the nucleus during the transcription of antioxidant response-related genes and subsequent transcriptions of other genes such as NADPH:quinone oxidoreductase-1 (*NQO1*), *NQO2*, and heme oxygenase 1 (*HO-1*) [12,13].

Many natural substances have been screened and studied for the development of antioxidant supplements. Methylsulfonylmethane (MSM) is a small molecule containing sulfur and a methyl group and is found in fruits, vegetables, grains, and milk [14]. MSM restores the activities of catalase, superoxide dismutase, glutathione reductase, and glutathione S-transferase, and can directly or indirectly reduce oxidative stress [15]. Therefore, MSM has historically been used in the treatment of inflammatory disorders such as arthritis, interstitial cystitis, acute allergic rhinitis, exercise-induced inflammation, autoimmune disease, and cancer [14].

Considering the limited number of studies on stress-induced metabolism in horse liver *in vivo*, alternative methods such as the use of *in vitro* systems are needed to study and confirm the effects of natural substances. In this study, we used fetal horse liver derived cells (FHLCs) *in vitro* and investigated the effects of MSM on the level of cellular oxidative stress induced by H_2_O_2_, to improve current understanding of hepatic oxidative stress responses.

## MATERIALS AND METHODS

### Tissue sampling

The experimental design was approved by the Pusan National University Institutional Animal Care and Use Committee (Approval Number: PNU-2015-0864). Tissue samples were collected via biopsies of the cerebrum, spinal cord, lungs, heart, liver, kidneys, and cecum of slaughtered horses.

### Cell culture and treatment

FHLCs were kindly provided by Professor Tae Sub Park from Seoul National University. The liver tissue of a 7-month-old fetal Jeju horse was used in this study. Cells were cultured in Dulbecco’s modified Eagle’s medium (Gibco, Grand Island, NY, USA) supplemented with 10% fetal bovine serum (Invitrogen, Carlsbad, CA, USA) and 1% antibiotic–antimycotic solution (Invitrogen, USA) at 37°C in a 5% CO_2_ incubator. After 3 to 4 days of incubation, at 80% confluence, cells were treated with various concentrations of H_2_O_2_ (Junsei, Tokyo, Japan) either in the presence or absence of MSM (Merck, Darmstadt, Germany). MSM was mixed with H_2_O_2_ in the medium and stored at 4°C for 1 h before treatment. Then, the cells were incubated for 4 h and washed twice with 1× phosphate-buffered saline (PBS) prior to RNA isolation.

### RNA isolation and reverse transcription-polymerase chain reaction

Total RNA was isolated using TRIzol reagent (Invitrogen, Karlsruhe, Germany), according to the manufacturer’s instructions. One microgram of RNA from each sample was used for reverse transcription with the SuperScript III First-Strand Synthesis System (Invitrogen, Germany). PRIMER3 software was used to design the primer sets. Information on the primers used in this study is provided in Table 1.

### Quantitative reverse transcription-polymerase chain reaction

NCBI (http://www.ncbi.nlm.nih.gov) and the Ensembl Genome Browser (www.ensembl.org) were used to retrieve gene sequence information. The primers for amplification of the genes (Table 1) were designed using PRIMER3 software. Quantitative reverse transcription-polymerase chain reaction (qRT-PCR) was performed using a thermal cycler (C1000 Thermal Cycler; Bio-Rad, Hercules, CA, USA) to measure the relevant expression of target genes in a 20 μL reaction volume composed of 2 μL diluted cDNA (20 ng/μL), 14 μL SYBR Green Master Mix (Bio-Rad, USA), and 1 μL each of diluted 5 pmol/μL forward and reverse primers. The qRT-PCR cycling conditions were as follows: initial denaturation at 94°C for 10 min, followed by 40 cycles of denaturation at 94°C for 30 s, annealing at 65°C for 30 s, and extension at 72°C for 30 s. All measurements were performed in triplicate, and the 2^−ΔΔCt^ method was used to determine relative gene expression. Relative expression of the target genes was normalized to the expression of glyceraldehyde-3-phosphate dehydrogenase.

### 3-(4,5-dimethylthiazol-2-yl)-2,5-diphenyltetrazolium bromide assay

Cell viability was assayed by measuring the amount of blue formazan generated from 3-(4,5-dimethylthiazol-2-yl)-2,5-diphenyltetrazolium bromide (MTT; Sigma-Aldrich, St. Louis, MO, USA) by the activity of mitochondrial dehydrogenases. The cells were resuspended in the medium one day before H_2_O_2_ treatment at a density of 2×10^5^ cells per well in 24-well culture plates. The control treatment consisted of a culture plate in which the medium was replaced with fresh medium containing dimethyl sulfoxide (DMSO). Cells were incubated with various concentrations of H_2_O_2_ (Junsei, Japan) for 4 h in either the presence or absence of MSM. MTT (0.5 mg/mL) was added to each well and incubated for 4 h at 37°C. The formazan product was dissolved by adding 200 μL DMSO to each well, and the absorbance was measured at 570 nm using a microplate reader (Tecan US Inc., Durham, NC, USA). All measurements were performed in triplicate and repeated at least three times.

### Oxidative stress analysis using dihydroethidium staining

H_2_O_2_-stimulated and unstimulated FHLCs were incubated in the presence or absence of MSM (100 mM or 200 mM) at 37°C for 4 h. The medium was removed, and 10 μM dihydroethidium (DHE; Sigma-Aldrich, USA) was added to the cells and incubated for an additional 1 h at 37°C. Then, the solution was removed, and the FHLCs were detached using 0.05% trypsin-ethylenediaminetetraacetic acid. Cells were washed with 1× PBS, resuspended in FACS buffer, and analyzed using a Muse Cell Analyzer (Merck, Germany).

## RESULTS AND DISCUSSION

### Characterization of FHLCs and expression of *CYP3A* isoforms

**Figure 1:**
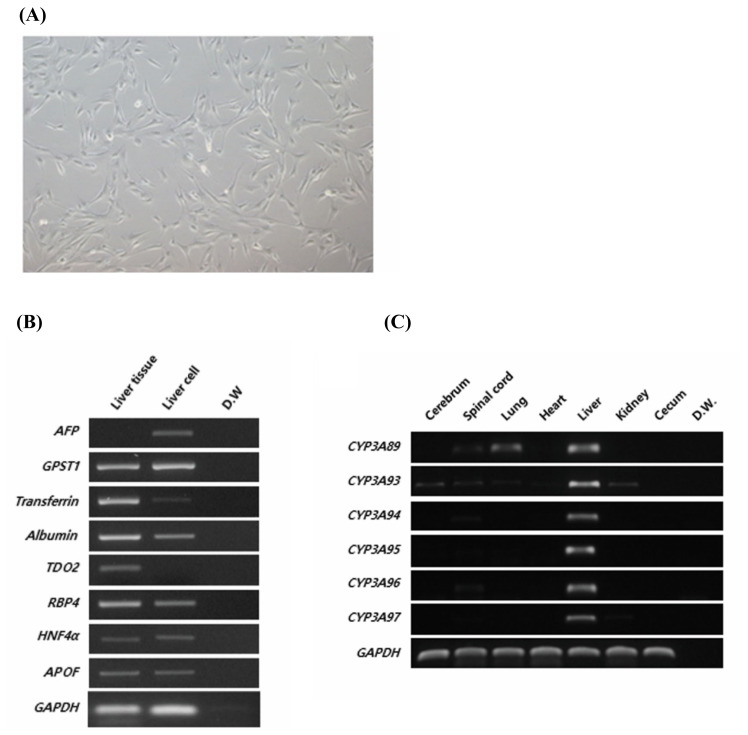
Characterization of horse liver-derived cells and validation of CYP3A family genes in different tissues of horse. (A) Morphology of horse liver-derived cells. Scale bar: 50 μm. (B) Liver marker expression analyzed using RT-PCR in horse liver tissue and liver-derived cells. *GAPDH* was used as the reference gene. (C) Expression of *CYP3A* genes in different horse tissues, namely, cerebrum, spinal cord, lungs, heart, liver, kidneys, and cecum, was analyzed using RT-PCR. *GAPDH* was used as the reference gene. The data are presented as one of three independent experiments. *CYP3A*, cytochrome P450 family 3 subfamily a; RT-PCR, reverse transcription-polymerase chain reaction; *AFP*, α-fetoprotein; *GSTP1*, glutathione S-transferase P; *TDO2*, tryptophan 2,3-dioxygenase; *RBP4*, retinol-binding protein 4; *HNF4α*, hepatocyte nuclear factor 4α; *APOF*, apolipoprotein F; *GAPDH*, glyceraldehyde 3-phosphate dehydrogenase; *CYP*, cytochrome P450.

We used FHLCs derived from the liver of a 7-month-old fetal Jeju horse (Figure 1A) and studied the expression of liver-specific markers (Figure 1B) to verify the lineage of cells. Fetal liver markers such as α-fetoprotein and glutathione S-transferase P were highly expressed in FHLCs, whereas the expression of liver markers such as transferrin and albumin, typically observed in livers of adult horses, were reduced. Other markers such as retinol-binding protein 4 (*RBP4*), hepatocyte nuclear factor 4α (*HNF4α*), and apolipoprotein F (*APOF*) were expressed at similar levels in both FHLCs and liver tissue in adult horses. It is noteworthy that tryptophan 2,3-dioxygenase was expressed in the adult liver but not in FHLCs.

**Figure 2:**
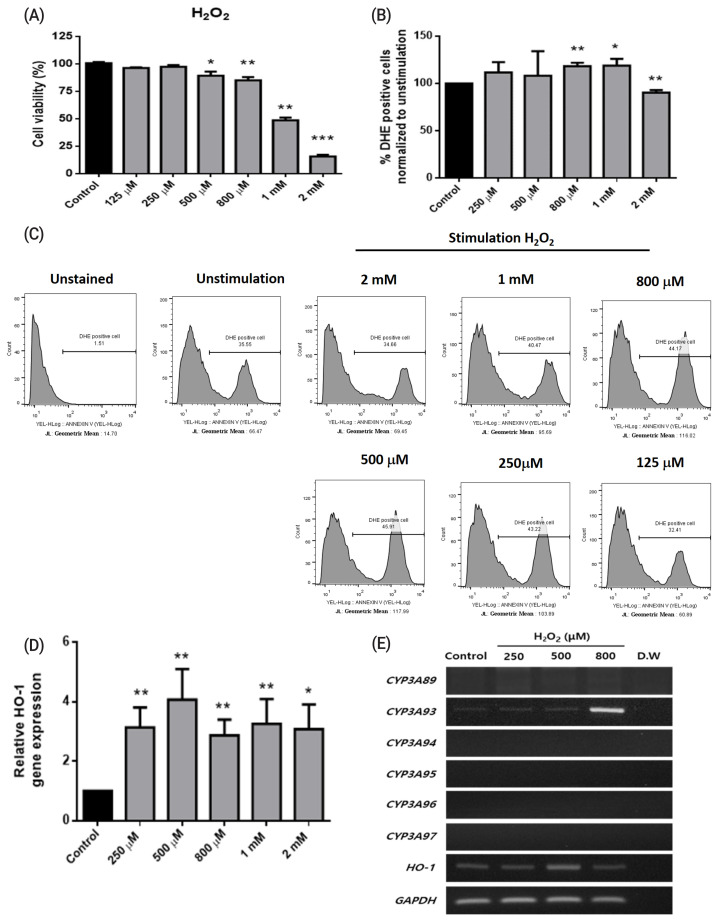
Effects of oxidative stress on horse liver-derived cells. (A) Cell viabilities measured using the 3-(4,5-dimethylthiazol-2-yl)-2,5-diphenyltetrazolium bromide (MTT) assay. (B) Effects of H2O2 treatment on the generation of reactive oxygen species analyzed using dihydroethidium (DHE) staining. (C) Histogram analysis of DHE-positive cells and geometric expression. (D) *HO-1* gene expression analyzed using real-time polymerase chain reaction (PCR). Black and gray bars represent data from control and H_2_O_2_ treatment, respectively. The data are presented as means±standard deviations of three independent experiments (* p<0.05, ** p<0.01, and *** p<0.001; unpaired Student’s *t*-test). (E) RT-PCR of *CYP3A* family gene expression after H_2_O_2_ treatment at 250, 500, and 800 μM for 4 h. Data are presented as one of three independent experiments. *CYP*, cytochrome P450; *HO-1*, heme oxygenase-1; *GAPDH*, glyceraldehyde 3-phosphate dehydrogenase.

To determine the expression patterns of the *CYP3A* gene family in horse, we designed specific primer sets to detect six *CYP3A* genes, namely, *CYP3A89*, *CYP3A93*, *CYP3A94*, *CYP3A95*, *CYP3A96*, and *CYP3A97*, in horse liver tissues (Table 1). All *CYP3A* genes were strongly expressed in liver tissue (Figure 1C). *CYP3A89* was weakly expressed in the spinal cord and lungs, and *CYP3A93* was differentially expressed in the cerebrum, spinal cord, liver, and kidneys. These results demonstrated that the *CYP3A* gene family was specifically expressed in liver tissues. In contrast to the adult liver tissue in which all *CYP3A* genes were expressed, only the expression of *CYP3A93* was detected in FHLCs (Figure 2E). In addition, the expression of the *CYP3A* gene family in FHLCs under oxidative stress was analyzed; the expression of *CYP3A93* was upregulated, but the expression of other *CYP3A* genes was not detected (Figure 2E).

Schmitz et al [8] annotate seven *CYP3A* genes in the horse genome and report that *CYP3A128P* is considered a pseudogene, while *CYP3A129* is expressed at undetectable levels in the liver. Previous studies have investigated the gene expression of CYP isozymes in horse liver tissues [13,16] and attempted functional characterization of these proteins [17, 18]. In this study, we observed that only *CYP3A93* was expressed in FHLCs, whereas all *CYP3A* genes were expressed in the adult liver of the horse. One possible explanation for the differential expression *CYP3A* genes and liver-specific genes in horse, e.g., *RBP4*, *HNF4α*, and *APOF*, may be attributed to the differentiation and maturation of hepatocytes. Zabulica et al [19] performed comparative experiments to provide a reliable guide for evaluating the differentiation of stem cells to hepatocyte-like cells using 60 genes expressed in the normal liver; they used a sample set of 17 fetal and 25 mature livers and revealed that the expression of *CYP3A* genes and other liver-marker genes are attributed to age [19]. Other studies have also shown that *CYP3A* mRNA expression follows a pattern very similar to that of *CYP3A* as revealed using immunostaining [20,21].

### Oxidative stress-induced expression of *CYP3A* family genes in FHLCs

To establish oxidative stress conditions, FHLCs were treated with different concentrations (125 μM, 250 μM, 500 μM, 800 μM, 1 mM, and 2 mM) of H_2_O_2_ for 4 h. Significant cytotoxicity was observed at concentrations more than 500 μM, and cell viability was significantly decreased after treatment with 1 mM and 2 mM H_2_O_2_ (p<0.05, Figure 2A). We performed DHE staining to measure the cellular state of oxidative stress; DHE used as a superoxide anion probe was oxidized to a fluorescent product (excitation, 480 nm; emission, 567 nm) [22]. DHE-positive cells, which were calculated using geometric means, were significantly increased at concentrations more than 800 μM and 1 mM H_2_O_2_, indicating that FHLCs treated with H_2_O_2_ were subjected to oxidative stress. We noted that the DHE positivity of FHLCs was significantly decreased at 2 mM H_2_O_2_, probably because of cell death (Figure 2B and Figure 2C). *HO-1* is one of the downstream genes of CYP3A93, and *HO-1* expression can be used as a marker of oxidative stress [13]. In FHLCs, *HO-1* expression significantly increased after treatment with H_2_O_2_ at concentrations ranging from 250 μM to 2 mM (Figure 2D).

To determine the effects of oxidative stress on the expression of *CYP3A* genes, FHLCs were treated with different concentrations of H_2_O_2_ (250, 500, and 800 μM). We found that, among the six *CYP3A* genes, only *CYP3A93* was upregulated under oxidative conditions and the highest expression was detected after treatment with 800 μM H_2_O_2_ (Figure 2E). For CYP3A93 and other CYP3A members, mRNA expression level and protein level were highly correlated in the horse liver and intestine [23]. It is reasonable to assume that oxidative stress upregulated the mRNA of *CYP3A93* in FHLCs and in turn led to an increase in CYP3A93 proteins as well as CYP3A93 enzymatic activity. Nonetheless, the relationship between mRNA level and protein synthesis of horse CYP3A93 under oxidative stress in FHLCs needs to be studied further.

### Effects of MSM on FHLCs under oxidative conditions

**Figure 3:**
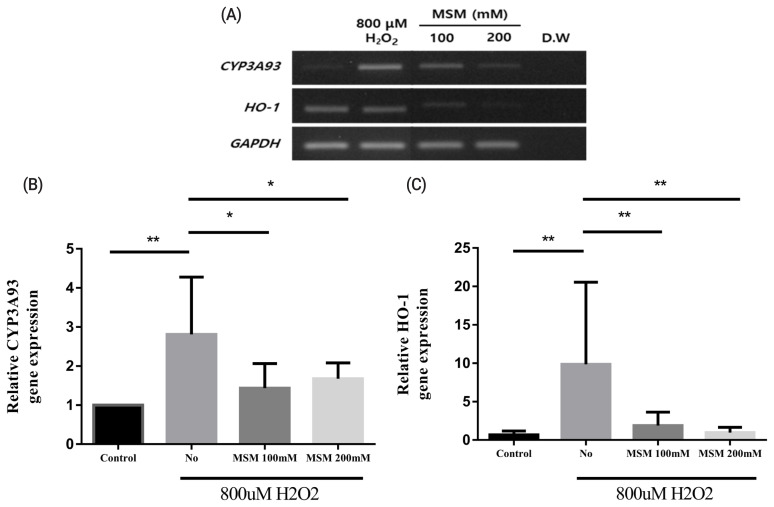
Effects of methylsulfonylmethane (MSM) on cell viability and reactive oxygen species generation in horse liver-derived cells under oxidative stress. (A) Percentage cell viability with MSM treatment at different concentrations (12.5, 25, 50, 100, and 200 mM). (B) Percentage cell viability with 100 or 200 mM MSM under oxidative stress conditions (800 μM H_2_O_2_). Reactive oxygen species levels were determined using dihydroethidium (DHE) staining and analyzed using flow cytometry. (C) Black and white histograms represent DHE-negative and DHE-positive cells, respectively. (D) Black, grey, and white bars represent the percentage of DHE-positive cells in the presence or absence of MSM treatment (100 or 200 mM) under oxidative stress (800 μM H_2_O_2_). Data are presented as means±standard deviations of three independent experiments (* p<0.05, ** p<0.01, and *** p<0.001; unpaired Student’s t-test). Cell viabilities were measured using 3-(4,5-dimethylthiazol-2-yl)-2,5-diphenyltetrazolium bromide (MTT) assay.

We tested whether the well-known antioxidant MSM could ameliorate oxidative stress in FHLCs and regulate *CYP3A93* expression. We found that the MSM (12.5, 25, 50, 100, and 200 mM) did not change the viability of FHLCs (Figure 3A). Next, we examined the effects of MSM in FHLCs under oxidative stress by exposure to 800 μM H_2_O_2_. Treatment with MSM did not restore the viability of FHLCs that was reduced due to oxidative stress (Figure 3B). Although low concentrations of MSM did not ameliorate oxidative stress, treatment with 200 mM MSM clearly reduced the percentage of DHE-positive cells (Figure 3C and 3D), suggesting that MSM blocked ROS generation under oxidative stress. It is noteworthy that MSM had the capacity to suppress ROS generation; however, it did not improve cellular viability. This finding suggested that MSM affected other pathways to regulate cell death and suppress ROS generation. Further studies are required to determine these involved pathways.

Next, we investigated the effects of MSM on *HO-1* and *CYP3A93* expression after treatment with 800 μM H_2_O_2_. *CYP3A93* expression decreased significantly after treatment with 100 and 200 mM MSM (Figure 4A, 4B). In addition, *HO-1* showed an expression pattern similar to that of *CYP3A93* (Figure 4A, 4C). These results indicated that *CYP3A93* expression could be used as a marker to determine oxidative stress in FHLCs. Nonetheless, it was unclear how MSM regulated *CYP3A93* expression and why reduced *CYP3A93* expression was correlated with reduced ROS generation in FHLCs. A previous study reveals that MSM reduces cortisol-induced stress through the expression of p53-mediated succinate dehydrogenase complex flavoprotein subunit A/hypoxanthine phosphoribosyltransferase 1 in racehorse muscle [24]. In addition, MSM has been shown to increase the expression of antioxidant enzymes such as peroxiredoxin-1, thioredoxin-1, and HO-1, which are normally induced by the activation of Nrf2 [25]. MSM can inhibit the transcriptional activity of NF-κB during ROS production [14]. Further studies are required to determine whether these pathways regulate MSM-mediated CYP3A93 suppression and ROS reduction in FHLCs.

## CONCLUSION

In this study, we investigated the expression of horse *CYP3A* genes in liver tissues of horse and FHLCs and found that *CYP3A93* was expressed in FHLCs. *CYP3A93* expression increased under oxidative conditions; however, the expression of other *CYP3A* genes did not increase. In FHLCs, MSM reduced ROS generation without affecting cell viability and suppressed the expression of *CYP3A93* and *HO-1*; these findings suggested that MSM can be used to decrease oxidative stress-induced hepatotoxicity in horses. Further investigation is required to clarify the mechanisms underlying these effects in FHLCs.

## Figures and Tables

**Table 1:** Primer sets used in this study

Primer name	Primer sequence (5′ to 3′)	Tm (°C)	Product size (bp)
GSTP1 F	AAGTTCCAGGACGGAGACCT	60	287
GSTP1 R	GAGATCTGGTTGCCCACAAT	-	-
Transferrin F	CCCAACCTGTGTCAACTGTG	60	219
Transferrin R	ACTGACTTCCGGGTGTTGTC	-	-
HNF4a F	GGTCGAGCTATGAGGACAGC	60	193
HNF4a R	ATGTACTTGGCCCACTCGAC	-	-
RBP4 F	ACCCTGCCAAGTTCAAGATG	60	170
RBP4 R	GGCAAACACGAAGGAGTAGC	-	-
Albumin F	GCACTTGCTGAACTGGTGAA	60	203
Albumin R	AGGCTGAGATGCTCGTGATT	-	-
AFP F	GCTGGCCTTATTATCGGACA	60	210
AFP R	TTGCAGTGCTACACCCTGAG	-	-
APOF F	CCCCTCTACCCAAGTTCCTC	60	289
APOF R	TCCTGCTCGTGTTCACAGTC	-	-
TDO2 F	TACCGCGATAACTTCCAAGG	60	107
TDO2 R	AAACCTGGTGTTCGTTCCAG	-	-
CYP3A89 F	TTCCTAAAGGGACAGTGGTGATG	58	209
CYP3A89 R	CCAGCTCCAAAGGGCAGGTA	-	-
CYP3A93 F	TCACCGAGCCTCAGAGTTTTG	58	302
CYP3A93 R	ATTGACGGTCCCATCTCTGG	-	-
CYP3A94 F	CTGATGTCCAGCAGAAGCTTCAG	58	142
CYP3A94 R	CTACCAGCAATTGGGAACAATC	-	-
CYP3A95 F	CCAAAGGGTCAACAGTGATGAT	58	147
CYP3A95 R	CAGTTCCAAAGGGCAGGTATGT	-	-
CYP3A96 F	CAAAGGAACAGTGGTGATG	58	152
CYP3A96 R	GGTCCATTTCCAAAGGGCATA	-	-
CYP3A97 F	TCCCAAAAGGACACTGGTGACT	58	149
CYP3A97 R	CGGTTCCAAAAGGCAGGTATAT	-	-
GAPDH F	GGTGAAGGTCGGAGTAAACG	60	106
GAPDH R	AATGAAGGGGTCATTGATGG	-	-

*GSTP1*, glutathione S-transferase P1; HNF4a, hepatocyte nuclear factor 4α; RBP4, retinol-binding protein 4; AFP, α-fetoprotein; APOF, apolipoprotein F; TDO2, tryptophan 2,3-dioxygenase; CYP3A, cytochrome P450 family 3 subfamily a; GAPDH, glyceraldehyde 3-phosphate dehydrogenase.

## References

[1] Guengerich FP (2006). Cytochrome P450s and other enzymes in drug metabolism and toxicity. AAPS J.

[2] Nelson DR (2006). Cytochrome P450 nomenclature, 2004. Methods Mol Biol.

[3] Zanger UM, Schwab M (2013). Cytochrome P450 enzymes in drug metabolism: regulation of gene expression, enzyme activities, and impact of genetic variation. Pharmacol Ther.

[4] Ding X, Kaminsky LS (2003). Human extrahepatic cytochromes P450: function in xenobiotic metabolism and tissue-selective chemical toxicity in the respiratory and gastrointestinal tracts. Annu Rev Pharmacol Toxicol.

[5] Schmitz A, Zielinski J, Dick B, Mevissen M (2014). *In vitro* metabolism of testosterone in the horse liver and involvement of equine CYPs 3A89, 3A94 and 3A95. J Vet Pharmacol Ther.

[6] Nakayama SMM, Ikenaka Y, Hayami A (2016). Characterization of equine cytochrome P450: role of CYP3A in the metabolism of diazepam. J Vet Pharmacol Ther.

[7] Liu YT, Hao HP, Liu CX, Wang GJ, Xie HG (2007). Drugs as CYP3A probes, inducers, and inhibitors. Drug Metab Rev.

[8] Schmitz A, Demmel S, Peters LM, Leeb T, Mevissen M, Haase B (2010). Comparative human-horse sequence analysis of the CYP3A subfamily gene cluster. Anim Genet.

[9] Deavall DG, Martin EA, Horner JM, Roberts R (2012). Drug-induced oxidative stress and toxicity. J Toxicol.

[10] Mishin V, Heck DE, Laskin DL, Laskin JD (2014). Human recombinant cytochrome P450 enzymes display distinct hydrogen peroxide generating activities during substrate independent NADPH oxidase reactions. Toxicol Sci.

[11] Zangar RC, Davydov DR, Verma S (2004). Mechanisms that regulate production of reactive oxygen species by cytochrome P450. Toxicol Appl Pharmacol.

[12] Kensler TW, Wakabayashi N (2010). Nrf2: friend or foe for chemoprevention?. Carcinogenesis.

[13] Loboda A, Damulewicz M, Pyza E, Jozkowicz A, Dulak J (2016). Role of Nrf2/HO-1 system in development, oxidative stress response and diseases: an evolutionarily conserved mechanism. Cell Mol Life Sci.

[14] Butawan M, Benjamin RL, Bloomer RJ (2017). Methylsulfonylmethane: applications and safety of a novel dietary supplement. Nutrients.

[15] Mohammadi S, Najafi M, Hamzeiy H (2012). Protective effects of methylsulfonylmethane on hemodynamics and oxidative stress in monocrotaline-induced pulmonary hypertensive rats. Adv Pharmacol Pharm Sci.

[16] Tyden E, Lofgren M, Hakhverdyan M, Tjalve H, Larsson P (2013). The genes of all seven CYP3A isoenzymes identified in the equine genome are expressed in the airways of horses. J Vet Pharmacol Ther.

[17] DiMaio Knych HK, DeStefano Shields C, Buckpitt AR, Stanley SD (2009). Equine cytochrome P450 2C92: cDNA cloning, expression and initial characterization. Arch Biochem Biophys.

[18] DiMaio Knych HK, McKemie DS, Stanley SD (2010). Molecular cloning, expression, and initial characterization of members of the CYP3A family in horses. Drug Metab Dispos.

[19] Zabulica M, Srinivasan RC, Vosough M (2019). Guide to the assessment of mature liver gene expression in stem cell-derived hepatocytes. Stem Cells Dev.

[20] Lee SJ, Hedstrom OR, Fischer K (2001). Immunohistochemical localization and differential expression of cytochrome P450 3A27 in the gastrointestinal tract of rainbow trout. Toxicol Appl Pharmacol.

[21] Cotreau MM, von Moltke LL, Beinfeld MC, Greenblatt DJ (2000). Methodologies to study the induction of rat hepatic and intestinal cytochrome P450 3A at the mRNA, protein, and catalytic activity level. J Pharmacol Toxicol Methods.

[22] Zhao H, Kalivendi S, Zhang H (2003). Superoxide reacts with hydroethidine but forms a fluorescent product that is distinctly different from ethidium: potential implications in intracellular fluorescence detection of superoxide. Free Radic Biol Med.

[23] Tyden E, Lofgren M, Pegolo S, Capolongo F, Tjalve H, Larsson P (2012). Differential gene expression of CYP3A isoforms in equine liver and intestines. J Vet Pharmacol Ther.

[24] Sp N, Kang DY, Kim DH (2020). Methylsulfonylmethane inhibits cortisol-induced stress through p53-mediated SDHA/HPRT1 expression in racehorse skeletal muscle cells: a primary step against exercise stress. Exp Ther Med.

[25] Withee ED, Tippens KM, Dehen R, Tibbitts D, Hanes D, Zwickey H (2017). Effects of methylsulfonylmethane (MSM) on exercise-induced oxidative stress, muscle damage, and pain following a half-marathon: a double-blind, randomized, placebo-controlled trial. J Int Soc Sports Nutr.

